# Effect of Aggregate Mass Fractal Dimension on Creep Behavior and Damage Mechanisms of Cemented Coal Gangue Backfill

**DOI:** 10.3390/ma19143110

**Published:** 2026-07-20

**Authors:** Yongjin Zhang, Cheng Li, Kangsheng Xue, Hui Yang, Zhen Lu

**Affiliations:** 1 School of Architectural Engineering, Kaili University, Kaili 556011, China; asd18798012397@163.com (Y.Z.); licheng_730@163.com (C.L.); yh7005@126.com (H.Y.); 2 State Key Laboratory of Intelligent Construction and Healthy Operation and Maintenance of Deep Earth Engineering, China University of Mining and Technology, Xuzhou 221116, China; ksxue@cumt.edu.cn; 3 School of Civil Engineering and Architecture, Guizhou Minzu University, Guiyang 550025, China

**Keywords:** cemented granular materials, coal gangue, fractal dimension, aggregate gradation, creep behavior, acoustic emission, damage evolution

## Abstract

**Highlights:**

- UCS, stepwise creep, AE, and SEM tests were conducted on fractal-graded CCGB.
- The best-performing aggregate mass fractal dimension (2.41) yields the highest empirical long-term strength among the tested groups.
- Fractal dimension regulates tensile–shear crack transition during creep.
- Microstructural evolution reveals the skeleton–interface coupling mechanism.
- A unified structural parameter linking gradation and creep is proposed.

**Abstract:**

Cemented coal gangue backfill (CCGB) is an important material for the resource utilization of mining solid waste, and its long-term stability is strongly affected by aggregate gradation. In this study, aggregate mass fractal dimension was used to characterize the particle size distribution of coal gangue, and four gradation schemes with different fractal dimensions were designed. Uniaxial compressive strength (UCS) tests, stepwise accelerated creep tests, acoustic emission (AE) monitoring, and scanning electron microscopy (SEM) observations were conducted to investigate strength, creep behavior, crack evolution, and damage mechanisms. The results show that P-wave velocity and UCS exhibit generally non-monotonic variations with fractal dimension, with relatively high values in the intermediate fractal-dimension range. The empirical long-term strengths for D=2.20, 2.41, 2.59, and 2.79 are 6.62, 8.83, 7.34, and 7.07 MPa, respectively. AE results indicate that shear cracking first decreases and then increases with fractal dimension, reaching the lowest proportion of 41.8% at D=2.41. SEM observations show that an intermediate fractal dimension improves skeleton continuity and interfacial integrity, thereby suppressing shear-related damage and delaying creep instability. These findings demonstrate that aggregate mass fractal dimension is an effective structural parameter for linking gradation characteristics, creep resistance, and damage evolution of CCGB.

## 1. Introduction

With the continuous exploitation of coal resources, large quantities of coal gangue are generated, making its efficient utilization a critical issue for sustainable and green mining practices [1,2,3,4,5]. The use of coal gangue as aggregate in cemented backfill materials provides an effective approach for large-scale solid waste reutilization while simultaneously controlling deformation of surrounding rock in underground excavations [6,7,8,9,10]. However, cemented backfill materials are typically subjected to long-term loading conditions during service, and their mechanical response exhibits pronounced rheological behavior, particularly creep deformation, which plays a crucial role in long-term stability [11,12]. Therefore, understanding the creep behavior of cemented coal gangue materials is of significant engineering importance.

The macroscopic mechanical performance of cemented backfill materials is governed by the combined effects of hydration products, pore structure, and aggregate skeleton [13,14,15,16,17]. Existing studies have predominantly focused on improving mechanical properties by optimizing binder type, dosage, water-binder ratio, and curing conditions [18,19,20,21]. International studies on cemented paste backfill have also shown that binder type, specimen composition, curing age, stress state, and microstructural evolution significantly affect the strength development and deformation behavior of cemented backfill materials [22,23,24]. However, such approaches are often constrained by material availability and economic considerations in practical engineering applications. Cemented backfill can be regarded as a porous particle–cement composite in which aggregate particles generally constitute the dominant mass fraction and are embedded within a hydrated cementitious matrix [25]. Consequently, the particle size distribution of aggregates significantly influences particle packing, skeleton formation, pore structure, and the mechanical performance of cemented backfill [26,27].

A considerable number of experimental studies have investigated the influence of aggregate gradation on the mechanical behavior of cemented materials; however, the reported conclusions remain inconsistent. For example, some studies have demonstrated a positive correlation between compressive strength and aggregate fineness [13,28], whereas others have indicated that moderate gradation yields optimal strength, or even that excessive fine particles may deteriorate mechanical performance [29,30,31]. These discrepancies highlight the complexity of aggregate structure effects. To address this issue, fractal approaches have been introduced in recent studies. For instance, fractal approaches have been increasingly used to characterize aggregate gradation, pore structure, rheological response, mechanical behavior, and permeability-related structural complexity in cemented backfill materials [32,33,34,35]. Nevertheless, a unified quantitative parameter capable of describing complex PSD characteristics and enabling transferable gradation design is still lacking. Furthermore, most existing research has focused on strength and instantaneous deformation, while time-dependent behaviors under realistic loading conditions, such as creep under multi-stage loading, remain insufficiently investigated, particularly in relation to structural parameters such as fractal dimension.

Previous studies on coal gangue-based cemented backfill have mainly focused on the effects of binder content, water-to-binder ratio, curing condition, activation method, aggregate particle size, and fine particle content on strength development and pore structure. These studies have confirmed that coal gangue can be used as a backfill aggregate and that its gradation affects the compactness and mechanical performance of cemented backfill. However, most existing studies evaluated gradation effects using single particle size ranges, fine particle content, or empirical grading descriptors, and the discussion was mainly limited to UCS or short-term mechanical behavior. The relationship among aggregate gradation, creep deformation, AE-based crack evolution, and microstructural damage mechanisms remains insufficiently clarified. Therefore, the present study differs from previous work by using aggregate mass fractal dimension as a unified structural parameter to link PSD characteristics with long-term creep response and damage evolution of cemented coal gangue backfill.

In view of these limitations, it is necessary to explore the time-dependent mechanical behavior of cemented coal gangue materials from the perspective of quantitative aggregate structure characterization. Fractal theory provides an effective framework for describing complex granular systems, and the fractal dimension of particle size distribution can comprehensively reflect the overall structural characteristics of aggregates. For granular materials and soils, fractal scaling has also been widely used to describe particle size distributions and to relate cumulative mass distribution to the fractal dimension of granular systems [36]. In this study, the mass fractal dimension (*D*) is introduced to characterize the PSD of coal gangue, and a series of aggregate gradation schemes with different fractal dimensions are constructed. Multi-stage loading creep tests are conducted to systematically investigate the influence of fractal characteristics on creep deformation behavior and steady-state creep rate. Furthermore, a simplified quantitative relationship between fractal dimension and creep parameters is established. The findings aim to elucidate the governing mechanisms linking aggregate structure to creep behavior, and to provide a theoretical basis for gradation optimization and long-term stability evaluation of cemented coal gangue materials.

The main contribution of this study lies in establishing aggregate mass fractal dimension as a quantitative structural parameter for linking particle size distribution, creep deformation, crack evolution, and microstructural damage mechanisms of cemented coal gangue backfill. Compared with conventional gradation descriptors such as single particle size, coarse-to-fine aggregate ratio, or empirical grading curves, the fractal dimension provides a unified parameter that reflects the overall distribution characteristics of aggregates over multiple particle size ranges. This enables a more direct comparison among different gradation schemes and provides a quantitative basis for optimizing backfill aggregate design from the perspective of long-term creep stability. Unlike conventional particle-packing theories that mainly aim to achieve maximum packing density or optimize fresh-state workability, the present fractal-dimension-based approach focuses on the structural role of aggregate gradation in time-dependent deformation and damage evolution. Therefore, the novelty of this study is not only the use of a fractal parameter to describe PSD, but also the establishment of a gradation–creep–damage linkage for cemented coal gangue backfill under sustained loading conditions. More specifically, previous fractal-based studies mainly used fractal parameters to describe pore structure, particle distribution, or geometric complexity, whereas the present study uses aggregate mass fractal dimension as an experimentally controllable gradation design parameter. The scientific contribution of this work is that the same structural parameter is linked simultaneously with UCS, steady-state creep rate, empirical long-term strength, Burgers rheological parameters, AE-based crack-mode evolution, and SEM-observed microstructural damage. Therefore, the proposed approach provides not only a description of PSD, but also a framework for interpreting gradation-controlled creep stability and damage mechanisms in cemented coal gangue backfill.

## 2. Materials and Methods

### 2.1. Raw Materials

**Figure 1:**
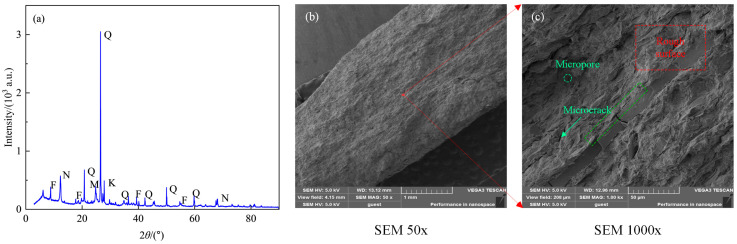
XRD pattern used for qualitative phase identification (**a**) and SEM images (**b**,**c**) of coal gangue.

The materials used for the cemented backfill specimens in this study mainly included coal gangue aggregate, cementitious binders (cement and fly ash), and purified water. The coal gangue was collected from a mining area in Xinjiang, China, and was used as aggregate after air-drying, crushing, and sieving. The crushed gangue particles exhibited irregular angular shapes and rough surfaces, which are favorable for developing strong mechanical interlocking between the aggregate and the cement matrix. The X-ray diffraction (XRD) pattern of the coal gangue was obtained using a D8 ADVANCE X-ray diffractometer (Bruker AXS GmbH, Karlsruhe, Germany) and scanning electron microscopy (SEM) (TESCAN GROUP, a.s., Brno, Czech Republic) images of coal gangue are shown in Figure 1, while the major oxide compositions of the raw materials adopted from Ref. [37] are listed in Table 1. The XRD results were used only for qualitative phase identification rather than quantitative phase analysis. Since Rietveld refinement was not performed, the relative peak intensities were not used to calculate mineral mass fractions. Therefore, the mineral phases of coal gangue were identified only according to the positions of the main diffraction peaks. The results indicate that the coal gangue mainly contains quartz, accompanied by feldspar and clay minerals. No quantitative mineral phase contents were derived from the XRD peak intensities. Quartz, with its high hardness and chemical stability, is beneficial for forming a stable granular skeleton within the cemented backfill material. SEM images further revealed that the gangue particles possessed irregular surface morphologies, with local micropores and microcracks. These microstructural characteristics enhance the mechanical interlocking between the aggregate and the cement matrix, while also potentially affecting the deformation evolution of the cemented backfill under long-term loading.

Although quantitative Rietveld refinement was not performed, the qualitative XRD results, together with the XRF chemical composition data, provide useful information for interpreting the mechanical and creep behavior of the coal gangue aggregate. The high SiO_2_ and Al_2_O_3_ contents obtained from XRF analysis are consistent with the presence of quartz and aluminosilicate minerals identified from the XRD peak positions. Quartz-rich particles can provide relatively rigid aggregate skeleton support because of their high hardness and stiffness, which contributes to stress transfer within the cemented matrix. However, the presence of clay minerals and pre-existing microcracks may increase local interfacial weakness and promote time-dependent deformation under sustained loading. During creep loading, these weak interfacial zones may become preferential locations for microcrack initiation, frictional sliding, and AE event generation. Therefore, the mineralogical and microstructural characteristics of coal gangue influence the creep response not only through aggregate stiffness, but also through the stability of the aggregate–matrix interfacial transition zone.

**Figure 2:**
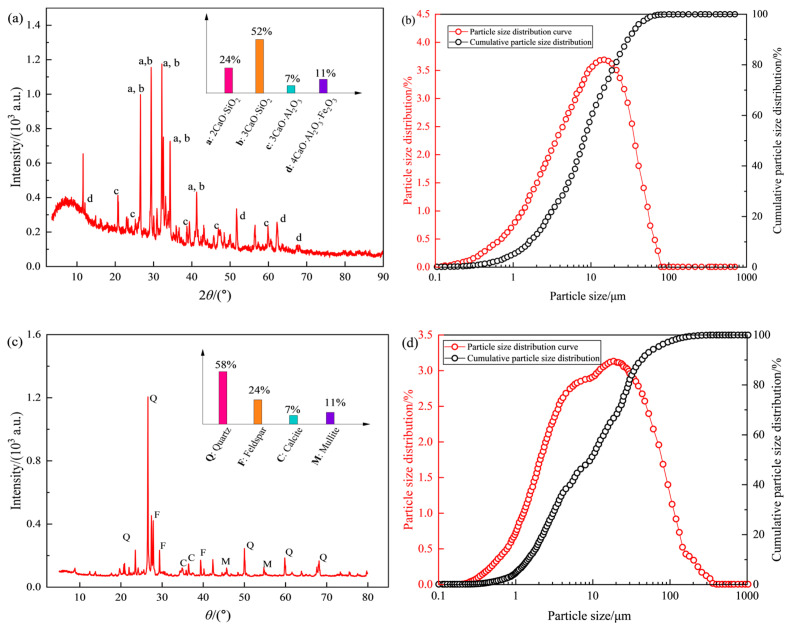
XRD patterns and particle size distributions of: (**a**) cement XRD; (**b**) cement PSD; (**c**) fly ash XRD; (**d**) fly ash PSD.

The chemical compositions of cement, fly ash, and coal gangue were determined by X-ray fluorescence (XRF) analysis [37]. Table 1 lists the major oxide components of the raw materials. The remaining fraction was reported as “Others and LOI”, which was calculated by difference and includes minor oxides and unquantified loss-on-ignition components. Ordinary Portland cement (P.O. 42.5) was used as the primary binder, and its XRD pattern and particle size distribution are shown in Figure 2a,b. The XRD pattern was used only to identify the main crystalline phases qualitatively. Fly ash was used as a partial replacement for cement, and its XRD pattern and particle size distribution are shown in Figure 2c,d. The particle size distribution results showed that the cement particles were relatively fine compared with the fly ash particles. Fly ash was used as a partial replacement for cement. According to the XRD and compositional results, the fly ash contained silica- and alumina-rich phases. Compared with cement, the fly ash particles were coarser overall and exhibited a wider particle size distribution range (Figure 2b). The incorporation of fly ash can reduce cement consumption and improve the particle composition of the binder system.

### 2.2. Specimen Preparation

**Figure 3:**
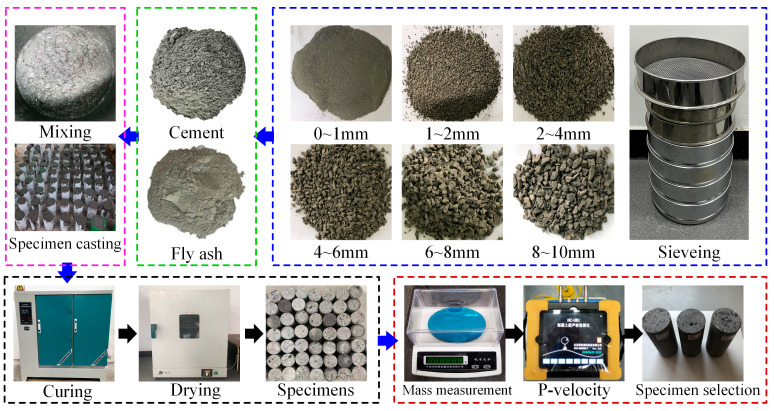
Specimen preparation procedure.

To carry out the mechanical tests on cemented coal gangue specimens, cylindrical samples were prepared in accordance with ISRM recommendations, with a diameter of 50 mm and a height-to-diameter ratio of 2:1 [38]. To minimize the influence of aggregate size effects on the test results, the maximum aggregate particle size was controlled to be less than 10 mm, approximately one-fifth of the specimen diameter. After crushing, the coal gangue was separated into different size fractions using standard sieves with apertures of 1.0, 2.0, 4.0, 6.0, 8.0, and 10.0 mm, and the particle size distribution was regulated according to mass fractal theory (Figure 3).

According to fractal theory, the number of particles larger than particle size *d*, denoted as *N*(>d), satisfies a power-law relationship [39,40,41]:

(1) $$N(>d)=C_{1}d^{-D}$$

where *D* is the fractal dimension used to describe the particle size distribution, and *C*_1_ is a constant. Furthermore, the cumulative number of particles smaller than or equal to particle size *d* can be expressed as:

(2) $$N(d)=N_{t}-N(>d)$$

where *N*_t_ is the total number of particles. By applying the boundary conditions *N*(*d*_min_) = 0 and *N*(*d*_max_) = *N*_t_, the cumulative particle number distribution function can be obtained as:

(3) $$f(d)=\frac{N(d)}{N_{t}}=\frac{d^{D}-d_{\min}^{D}}{d_{\max}^{D}-d_{\min}^{D}}$$

where *d*_min_ and *d*_max_ are the minimum and maximum particle sizes, respectively.

In practical mix design, aggregate gradation is usually expressed in terms of mass fraction. Assuming that the density of coal gangue particles is approximately constant over different size ranges, the mass of a single particle, *m*(*d*), can be related to its particle size *d* by:

(4) $$m(d)=C_{2}d^{3}$$

where *C*_2_ is a constant. The mass increment of aggregate particles within a certain particle size interval [d,d+dd] can then be expressed as:

(5) dM(d)=m(d)dN(d)

By substituting Equation (4) into Equation (5) together with Equation (1), and integrating over the particle size interval $\left[d_{\min},d\right]$, the cumulative mass of aggregate particles smaller than d can be obtained as:

(6) $$M(d)=C_{3}(d^{3-D}-d_{\min}^{3-D})$$

where *C*_3_ is a constant.

The cumulative mass fraction of particles smaller than *d*, denoted as *P*(*d*), is defined as the ratio between the cumulative mass *M*(*d*) and the total mass *M*_t_. By using the boundary conditions *P*(*d*_min_) = 0 and *P*(*d*_max_) = 1, the mass fractal model can be derived as:

(7) $$P(d)=\frac{M(d)}{M_{t}}=\frac{d^{3-D}-d_{\min}^{3-D}}{d_{\max}^{3-D}-d_{\min}^{3-D}}$$

Furthermore, when $d_{\min}\ll d_{\max}$, $d_{\min}^{3-D}≃0$, Equation (7) can be simplified as:

(8) $$P(d)={\left(\frac{d}{d_{\max}}\right)}^{3-D}$$

For a given particle size interval [*d_i_*, *d_i_*_+1_], the corresponding mass fraction can be calculated as:

(9) $$\omega_{i}=P(d_{i+1})-P(d_{i})$$

where P(d) is the cumulative mass fraction of particles smaller than d, $d_{min}$ and $d_{max}$ are the minimum and maximum particle sizes, respectively, $w_{i}$ is the mass fraction of particles within the i-th size interval, and D is defined in this study as the aggregate mass fractal dimension.

**Figure 4:**
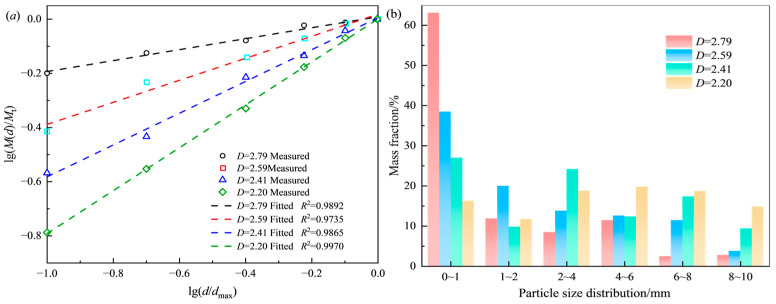
Aggregate mass fractal gradation design: (**a**) fractal fitting of the aggregate particle-size distributions; (**b**) mass fractions of coal gangue aggregates in different particle-size intervals for *D* = 2.20, 2.41, 2.59, and 2.79.

In this study, D is finally expressed and discussed as the aggregate mass fractal dimension because the designed gradation is controlled by the cumulative mass fraction of coal gangue particles. Based on Equation (8), the fractal dimensions of aggregate particles under different particle size distributions can be determined, as shown in Figure 4a. Figure 4b presents the mass fraction distributions in different particle size intervals. It can be seen that the mass fraction of coarse aggregates increases with decreasing fractal dimension, whereas the mass fraction of fine aggregates increases with increasing fractal dimension.

The four aggregate mass fractal dimensions were selected to represent a broad gradation range from coarse-particle-dominated to fine-particle-rich distributions under the available particle size intervals of 1–10 mm. Specifically, D=2.20 represents a relatively coarse gradation with a higher proportion of large particles, D=2.41 and D=2.59 represent intermediate gradations, and D=2.79 represents a relatively fine gradation with a higher proportion of small particles. These four levels were designed to capture the non-monotonic influence of gradation on strength, creep, and damage evolution while maintaining the same binder content, water-to-binder ratio, aggregate content, and curing condition. It should be noted that the term “optimal fractal dimension” in this study refers to the best-performing gradation within the present experimental range, rather than a universal optimum for all cemented backfill systems. More fractal-dimension levels and repeated creep tests are still needed in future studies to further refine the optimal gradation interval.

The proposed mass fractal model should be distinguished from the Modified Andreasen and Andersen (MAA) particle packing model. The MAA model is generally used to construct a target particle size distribution for achieving high packing density, and its distribution modulus is selected to optimize the packing state of solid particles in cementitious mixtures. In contrast, the mass fractal model used in this study was not intended to design a maximum-packing-density curve. Instead, the aggregate mass fractal dimension was introduced as a structural descriptor of the coal gangue aggregate PSD. Its purpose was to quantify the overall coarse-to-fine particle distribution and further relate this distribution to creep deformation, empirical long-term strength, AE-based crack evolution, and microstructural damage mechanisms. Therefore, while both methods describe continuous particle size distributions, the MAA model mainly serves mixture packing optimization, whereas the present fractal-dimension-based method focuses on the structural interpretation of gradation-controlled creep stability.

Based on the mass fractions in different particle size intervals, specimens with different aggregate mass fractal dimensions were prepared as follows. First, cement and fly ash were fully mixed with water to form a homogeneous cementitious slurry (Figure 3). The slurry was then mixed with coal gangue aggregates proportioned according to the designed fractal gradation so that the binder could uniformly coat the aggregate particles and form a homogeneous mixture. The fresh mixture was poured into molds for preparing standard cylindrical specimens with dimensions of Φ50 mm × 100 mm. Vibration was applied to ensure uniform distribution of the aggregate and slurry and to eliminate internal air bubbles, thereby improving specimen homogeneity. After molding, the specimens were left at room temperature until final setting, then demolded and cured in a standard curing chamber at 25 °C and 95% relative humidity. After 27 days of curing, the specimens were used for subsequent mechanical testing.

For all specimens with different aggregate mass fractal dimensions, the mass fractions of coal gangue aggregate, fly ash, and cement were maintained at 65%, 4%, and 16%, respectively, with the remaining 15% being water. Therefore, the total binder content, defined as the sum of cement and fly ash, was 20% by mass. The water-to-binder ratio (W/B) was 0.75, and the cement-to-fly ash ratio (C/FA) was 4.0. The solid mass concentration of the fresh backfill mixture, also referred to as slurry concentration, was calculated as the ratio of total solids to the total mixture mass and was 85%. The coal gangue aggregate was used in an air-dried condition after crushing and sieving. To improve the reproducibility of the specimen preparation procedure, the complete mix design is summarized in Table 2.

For each aggregate gradation group, more than five cylindrical specimens were initially prepared. Before mechanical testing, specimens with visible surface defects, edge damage, large voids, or obvious dimensional deviations were discarded. This screening procedure was conducted before UCS testing and was based only on specimen appearance, dimensional quality, mass, and P-wave velocity, rather than on the measured strength results. Five valid specimens were finally used for UCS testing in each aggregate gradation group. No maximum or minimum UCS value was excluded after testing; all valid UCS results were retained and included in the statistical analysis. The mean value and standard deviation were used to represent the strength level and data dispersion of each group. For fractal dimensions of 2.20, 2.41, 2.59, and 2.79, the average peak strengths were 10.40, 13.49, 12.72, and 11.21 MPa, respectively. These results provided the basis for the subsequent creep tests.

### 2.3. Experimental Equipment and Testing Scheme

**Figure 5:**
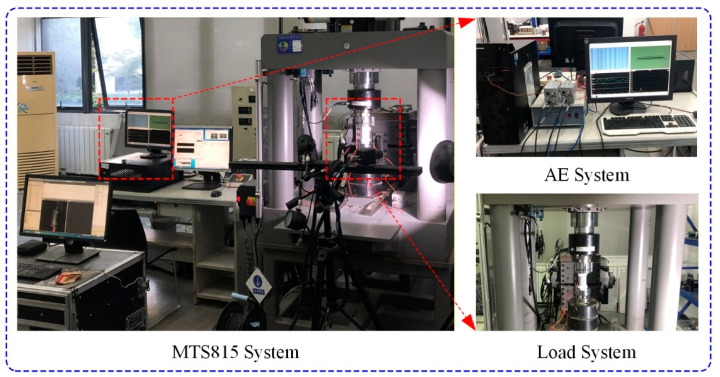
Main experimental instruments and testing system.

The main experimental equipment included an MTS815 electro-hydraulic servo-controlled rock testing system (MTS Systems Corporation, Eden Prairie, MN, USA) and an acoustic emission (AE) monitoring system, as shown in Figure 5. The maximum axial load capacity of the MTS816 system is 1700 KN, and the axial loading rate can be controlled within the range of 10^−5^ to 1 mm/s. This system can be used for both uniaxial compression and creep tests as required.

In this study, the creep behavior was investigated using a stepwise accelerated creep loading scheme. The purpose of this scheme was to compare the relative creep resistance and damage evolution of specimens with different aggregate mass fractal dimensions under the same loading path, rather than to directly reproduce the full long-term deformation of backfill materials during engineering service. The loading rate was set to 0.02 kN/s, and the load was increased by 4 kN, corresponding to a stress increment of approximately 2 MPa, at each stage. Each loading stage was maintained for 2 h before the next load increment was applied, until creep failure occurred. This holding duration was adopted because the axial creep rate of the specimens generally decreased and approached a quasi-steady state within this period under low and medium stress levels. This was confirmed by the creep-rate curves, in which the deformation rate became relatively stable before the next loading step for most specimens at low and medium stress levels. Therefore, the 2 h stage duration was considered suitable for extracting comparative quasi-steady creep rates under the present accelerated loading condition. It should be noted that this test scheme differs from conventional long-duration constant-stress creep tests, and the obtained creep parameters and empirical long-term strength should be interpreted as comparative accelerated creep indices.

For AE monitoring, four Nano30 sensors were attached tightly to the specimen surface using a coupling agent. The AE threshold was set to 40 dB in order to capture fracture signals during the creep loading process. The AE parameters were further analyzed using the rise angle–average frequency (RA–AF) method to identify the dominant cracking mode during creep loading [42]. The RA value was defined as the ratio of rise time to amplitude, while the AF value was defined as the ratio of AE counts to duration. In general, tensile cracking is characterized by relatively low RA and high AF values, whereas shear cracking is characterized by relatively high RA and low AF values. Because the absolute values of RA and AF are affected by sensor type, threshold setting, specimen geometry, coupling condition, and signal attenuation, a normalized RA–AF classification criterion was adopted in this study. Specifically, the RA and AF values were normalized as $RA_{n}=RA/RA_{max}$ and $AF_{n}=AF/AF_{max}$, where $RA_{n}$ and $AF_{n}$ are the normalized RA and AF values, respectively, and $RA_{max}$ and $AF_{max}$ are the maximum RA and AF values of the effective AE events used for classification. The boundary line $RA_{n}=AF_{n}$ was used to distinguish the cracking modes. AE events with $AF_{n}>RA_{n}$ were classified as tensile cracking events, whereas AE events with $RA_{n}>AF_{n}$ were classified as shear cracking events. This classification was used to calculate the relative proportions of tensile and shear cracking events under different aggregate mass fractal dimensions.

The experimental data were processed as follows. For the replicated physical and mechanical tests, the mean value and standard deviation (SD) were used to evaluate the repeatability and dispersion of the experimental results. Error bars in the relevant figures represent ±1 SD. Considering the limited number of aggregate mass fractal-dimension levels and the inherent heterogeneity of cemented coal gangue backfill, the statistical interpretation in this study focused on repeated-test dispersion and the consistency of variation trends rather than on determining a universal optimum through hypothesis testing. For the stepwise accelerated creep tests, the creep curves were mainly used for comparative analysis of different gradation groups under the same loading path. Considering the relatively limited number of creep specimens, the creep results were interpreted as comparative accelerated creep responses rather than as fully statistically validated long-term creep properties. The limitation associated with the relatively limited number of creep specimens and the accelerated loading procedure is further discussed in the subsequent sections.

## 3. Results and Discussion

### 3.1. Basic Physical and Mechanical Properties

**Figure 6:**
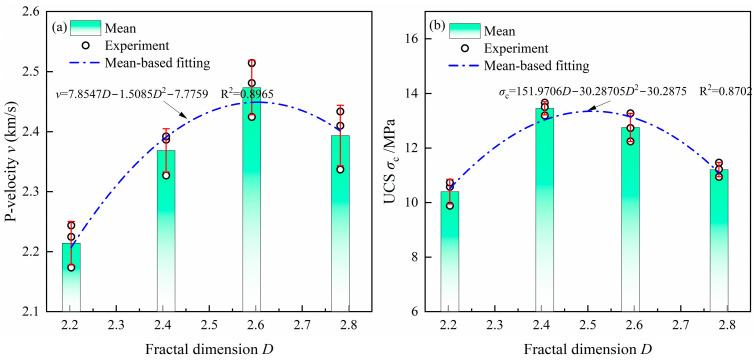
Variation in P-wave velocity (**a**) and uniaxial compressive strength (**b**) with fractal dimension.

To investigate the influence of aggregate particle size distribution on the fundamental physical and mechanical properties of cemented coal gangue backfill, ultrasonic wave velocity and uniaxial compressive strength (UCS) were measured for specimens with different fractal dimensions of aggregate gradation. The corresponding results are presented in Figure 6.

As shown in the figure, both ultrasonic wave velocity and UCS exhibit similar variation trends with increasing fractal dimension, characterized by an initial increase followed by a decrease. This non-monotonic behavior indicates the existence of an optimal fractal dimension range for the aggregate gradation.

When the fractal dimension is within a moderate range, both P-wave velocity and UCS reached relatively high values within the intermediate fractal-dimension range. In particular, the specimen with D=2.41 exhibited the highest UCS and overall favorable mechanical performance among the tested groups. This result suggests that an appropriate particle size distribution facilitates the formation of a more stable and compact aggregate skeleton, thereby enhancing the overall load-bearing capacity of the cemented backfill.

In contrast, when the fractal dimension is relatively low, the proportion of coarse particles is high. Although such a structure can provide a certain degree of skeletal support, the larger inter-particle voids limit the effectiveness of cementation, resulting in reduced mechanical performance. On the other hand, when the fractal dimension is excessively high, the increased content of fine particles weakens the skeleton effect and reduces structural stability, which is also unfavorable for strength development.

The repeated physical and mechanical tests showed consistent variation trends among the different gradation groups. After specimen quality screening based on appearance, mass, and P-wave velocity, all valid test results were used to determine the representative values of each group. The non-monotonic changes in P-wave velocity and UCS indicate that the intermediate aggregate mass fractal dimension produced a more favorable gradation structure among the tested groups. Nevertheless, considering the limited number of specimens and the inherent heterogeneity of cemented coal gangue backfill, the optimal fractal dimension is interpreted as the best-performing gradation within the present experimental range rather than as a universal optimum.

The error bars in Figure 6 represent the dispersion of repeated measurements. The repeated UCS and P-wave velocity tests showed consistent variation trends among the different aggregate gradation groups, indicating acceptable repeatability of the specimen preparation and testing procedures. Although some scatter was observed due to the inherent heterogeneity of cemented coal gangue backfill, both P-wave velocity and UCS exhibited a similar non-monotonic trend with increasing aggregate mass fractal dimension. Therefore, the improved performance at D=2.41 should be interpreted as the best-performing gradation among the tested groups rather than as a universal optimal fractal dimension. More repeated tests and additional fractal-dimension levels are still needed to further verify the statistical robustness of the optimal gradation range. Therefore, the present results should be regarded as evidence of a consistent experimental trend under the tested conditions, rather than as a statistically exhaustive determination of a universal optimum.

To quantitatively evaluate the relationship between P-wave velocity and UCS, a correlation analysis was conducted using the average values of the four aggregate gradation groups. The Pearson correlation coefficient between P-wave velocity and UCS was 0.661, indicating a moderate positive correlation. A linear fitting relationship was obtained as $UCS=8.542V_{p}-8.226$, with $R^{2}=0.436$, where $V_{p}$ is the P-wave velocity in km/s and UCS is expressed in MPa. This result indicates that specimens with higher P-wave velocity generally tended to exhibit higher compressive strength, which can be attributed to improved compactness, better aggregate–matrix bonding, and fewer internal defects. However, the correlation was not strong enough to support P-wave velocity as an independent predictor of UCS. This is because P-wave velocity mainly reflects wave propagation stiffness and internal compactness, whereas UCS is also affected by local interfacial defects, aggregate skeleton continuity, and crack coalescence behavior during loading. Therefore, the slight difference between the peak P-wave velocity and the peak UCS is attributed to their different physical meanings rather than manual error. P-wave velocity was used in this study as a nondestructive auxiliary indicator for evaluating specimen quality and relative mechanical performance.

### 3.2. Creep Mechanical Behavior of Cemented Specimens

#### 3.2.1. Axial Creep Deformation Characteristics

**Figure 7:**
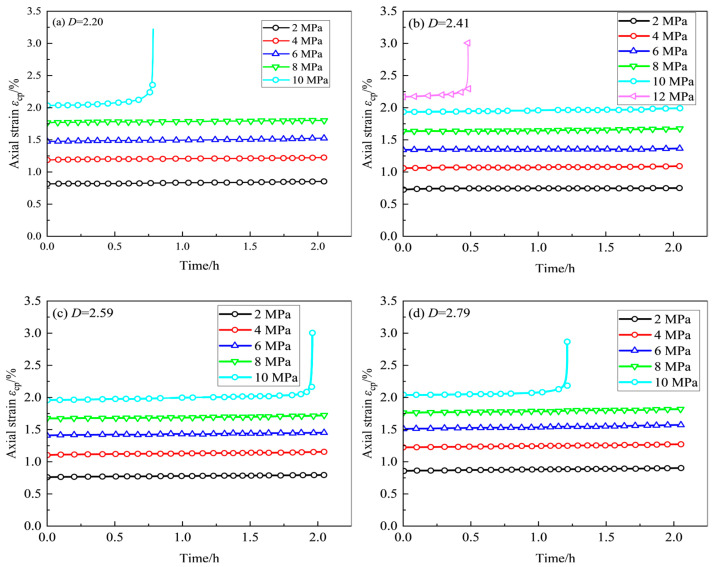
Axial creep–time curves of cemented coal gangue backfill specimens with different aggregate mass fractal dimensions: (**a**) *D* = 2.20, (**b**) *D* = 2.41, (**c**) *D* = 2.59, and (**d**) *D* = 2.79. Arrows indicate the accelerated creep stage before creep instability.

Based on the Boltzmann superposition principle, the creep–time curves under different stepwise stress levels were obtained, as shown in Figure 7. It can be observed that, at low stress levels (2–4 MPa), the axial strain of all specimens with different fractal dimensions increased rapidly immediately after loading, and then the strain growth rate decreased significantly. The creep deformation gradually approached a stable state within a relatively short period, exhibiting a typical decelerating creep behavior.

As the stress level increased to 6–8 MPa, the creep deformation began to increase at an approximately constant rate, indicating the onset of the steady-state creep stage. At the final stress level, some specimens successively experienced three typical stages, namely decelerating creep, steady-state creep, and accelerating creep. In this stage, the axial strain increased rapidly within a short time, and obvious creep instability failure occurred.

At the same time, clear differences were observed in the creep evolution behavior of specimens with different fractal dimensions at high stress levels. For example, when the fractal dimension was *D* = 2.20, obvious accelerating creep appeared at a stress level of 10 MPa, and the time to creep failure was relatively short. For the specimen with *D* = 2.79, although the creep deformation remained relatively small at low stress levels, the duration of the steady-state creep stage was significantly shortened at high stress levels, and the specimen also entered the accelerating creep stage at an earlier time. In contrast, specimens with intermediate fractal dimensions, such as *D* = 2.41 and *D* = 2.59, showed relatively slower creep deformation development under the same stress level. Their steady-state creep stages were markedly prolonged, and the occurrence of creep failure was significantly delayed.

As highlighted by the arrows in Figure 7, the accelerated creep stage occurs near the final loading level and is characterized by a rapid increase in axial strain before creep instability. In addition, the specimens with *D* = 2.41 and *D* = 2.59 generally exhibited creep instability at higher stress levels, whereas specimens with excessively low or high fractal dimensions failed under relatively lower stress levels. Combined with the static mechanical test results presented above, this finding suggests that specimens with intermediate fractal dimensions possess a more favorable aggregate skeleton structure, which can delay internal structural adjustment and damage evolution during stepwise loading, thereby leading to a more stable creep response compared with the other gradation conditions.

#### 3.2.2. Axial Creep Rate Characteristics

**Figure 8:**
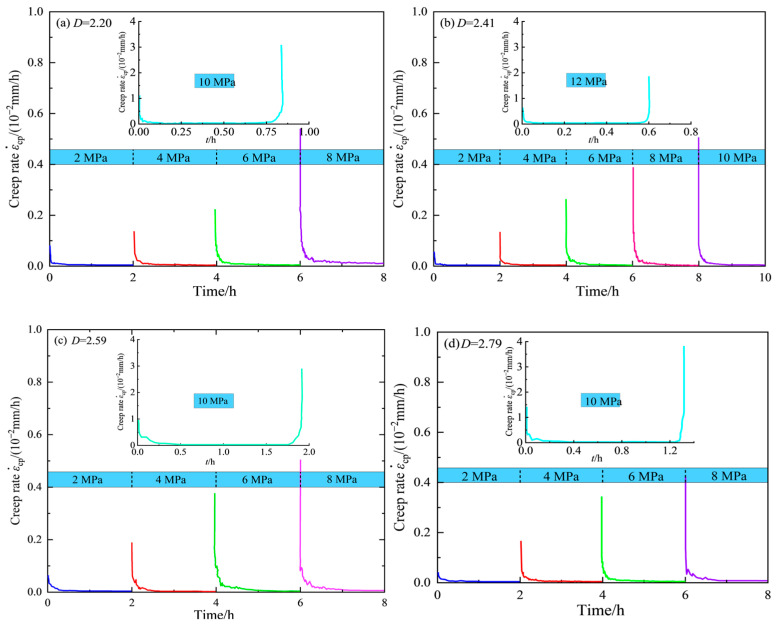
Axial creep rates of cemented specimens with different aggregate mass fractal dimensions.

Figure 8 presents the steady-state creep rates ${\dot{\varepsilon }}_{\mathrm{cp}}$ of the cemented specimens under stepwise loading conditions. It can be observed that, under different stress levels, the axial creep rates of specimens with different fractal dimensions generally increase with increasing stress. However, the magnitude of variation and the evolution patterns show significant differences among the specimens.

At low stress levels, the creep rate is relatively high at the initial stage of loading and then gradually decreases to a stable value. For example, for the specimen with a fractal dimension of *D* = 2.20 under a stress level of 2 MPa, the creep rate decreased from an initial value of 4.65 × 10^−4^ mm/h to 2.06 × 10^−5^ mm/h after 2 h of loading. Compared with the initial creep rate, the steady-state creep rates at low stress levels are generally small for all specimens, and the slopes of the curves are relatively similar, indicating that the influence of fractal dimension on creep rate is not significant at this stage.

As the stress level increases to a moderate range, the differences in steady-state creep rates ${\dot{\varepsilon }}_{\mathrm{cp}}$ among specimens with different fractal dimensions become more pronounced, suggesting that the regulatory effect of fractal dimension on creep behavior begins to emerge. For instance, at a stress level of 8 MPa, the specimen with *D* = 2.41 exhibits a lower steady-state creep rate ${\dot{\varepsilon }}_{\mathrm{cp}}$ (2.25 × 10^−5^ mm/h) compared with those with *D* = 2.20 (8.17 × 10^−5^ mm/h) and *D* = 2.79 (4.36 × 10^−5^ mm/h).

At the final loading stage, the creep rate shows a characteristic evolution pattern: a rapid decrease at the initial stage, followed by a relatively short steady-state stage, and then a sharp increase associated with accelerating creep. Compared with the creep rates observed at lower stress levels, the duration of the steady-state stage becomes significantly shorter prior to creep failure. For example, when *D* = 2.59 under a stress level of 10 MPa, the creep rate ${\dot{\varepsilon }}_{\mathrm{cp}}$ decreased from 97.6 × 10^−4^ mm/h to a stable value within 1.8 h, and then rapidly increased to 311 × 10^−4^ mm/h.

These results indicate that the axial creep rate exhibits a non-monotonic variation with fractal dimension, first decreasing and then increasing, implying the existence of an optimal fractal dimension range corresponding to a relatively low steady-state creep rate. This phenomenon suggests that an appropriate aggregate particle size distribution promotes the formation of a more stable load-bearing skeleton within the cemented material, thereby suppressing particle rearrangement and microstructural evolution under sustained loading. As a result, the material maintains a lower deformation rate during the steady-state creep stage. This characteristic provides an important basis for understanding the long-term deformation behavior and creep instability of cemented backfill materials.

#### 3.2.3. Long-Term Strength Characteristics

Long-term strength refers to the maximum stress level that a material can sustain while remaining stable under prolonged stress or deformation. It is an important mechanical parameter for evaluating the long-term stability of materials. In engineering practice, long-term strength is commonly determined by indirect methods, among which the steady-state creep rate method is widely used. Based on the foregoing analysis of axial creep rate, the long-term strengths of cemented specimens with different aggregate mass fractal dimensions were determined in this study using the steady-state creep rate approach.

In this study, the empirical long-term strength was determined from the relationship between steady-state creep rate and applied stress. For each fractal dimension, the quasi-steady-state creep rate at each stress level was first extracted from the approximately stable segment of the corresponding creep-rate curve. The stress-dependent creep-rate data were then fitted in the low-stress and high-stress regimes, respectively. The intersection of the two fitted curves was taken as the critical stress at which the creep response changed from slow stable growth to accelerated growth, and this stress was defined as the empirical long-term strength. Because this value was obtained from stepwise accelerated creep tests and curve fitting rather than from long-duration constant-stress creep tests, it inevitably contains uncertainty related to stress interval, stage duration, and fitting method. Therefore, the obtained long-term strength values should be interpreted as comparative empirical indices for different gradation groups rather than absolute long-term strength limits.

Mathematically, the low-stress and high-stress regimes were fitted separately using exponential functions, denoted as $f_{1}(\sigma )$ and $f_{2}(\sigma )$, respectively. The empirical long-term strength $\sigma_{L}$ was then obtained by solving:

(10) $$f_{1}\left(\sigma_{L}\right)=f_{2}\left(\sigma_{L}\right)$$

The solution located within the transition stress interval between stable creep and accelerated creep was selected as the empirical long-term strength. When no closed-form analytical solution was available, the intersection was obtained numerically from the fitted curves. This procedure ensured that the determined long-term strength corresponded to the stress level at which the steady-state creep rate began to increase rapidly.

Several methods have been used in the literature to estimate the long-term strength or creep threshold of cemented geomaterials, including long-duration constant-stress creep tests, isochronous stress–strain curve methods, creep-rate transition methods, and constitutive-model-based extrapolation. Among these methods, long-duration constant-stress creep tests can provide more direct long-term strength information, but they require a large number of specimens and long testing periods. The isochronous stress–strain method is also useful for identifying stress thresholds, but it requires sufficient creep data at multiple sustained loading durations. In the present study, the steady-state creep-rate transition method was adopted because the tests were designed as stepwise accelerated creep tests for comparative evaluation among different aggregate gradations. Therefore, the obtained long-term strength values should be regarded as empirical comparative indices rather than absolute long-term strength limits. The Burgers-model-based fitting in Section 3.2.4 was further used as an auxiliary comparison to evaluate the relative creep resistance of different gradation groups.

**Figure 9:**
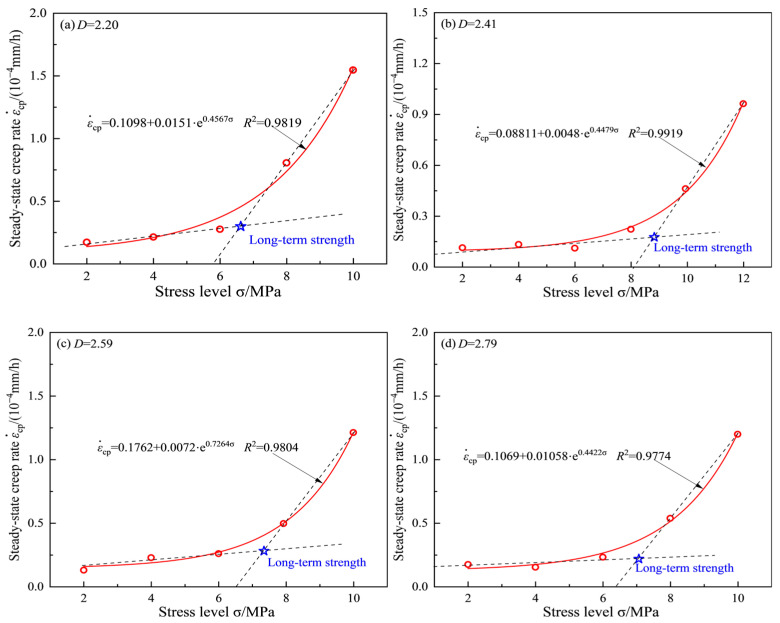
Relationship between steady-state creep strain rate and stress level: (**a**) D=2.20; (**b**) D=2.41; (**c**) D=2.59; (**d**) D=2.79. Red open circles represent the experimental quasi-steady-state creep rates, red solid curves represent the exponential fitting results, dashed lines represent the fitted low-stress and high-stress regimes, and blue stars indicate the empirical long-term strengths determined from the intersections of the two regime-fitting curves.

Figure 9 shows the variation in steady-state creep rate with stress level for specimens with different fractal dimensions of aggregate particle size distribution. It can be seen that the steady-state creep rate of all specimens exhibits a nonlinear increasing trend with increasing stress level, which can be generally described by an exponential function, as expressed in Equation (9). According to the fitting results, the contribution of the exponential term is relatively small in the low-stress range, and the steady-state creep rate increases slowly with stress. However, when the stress level exceeds a certain threshold, the exponential term begins to dominate, and the steady-state creep rate increases rapidly.

Further analysis based on piecewise fitting indicates that the intersection point between the fitted curves in the low-stress and high-stress regimes corresponds to the critical stress level at which the steady-state creep rate changes from slow growth to rapid acceleration. This critical stress can therefore be taken as the long-term strength criterion for the cemented material.

Specifically, for specimens with fractal dimensions of 2.20, 2.41, 2.59, and 2.79, the corresponding long-term strengths are 6.62, 8.83, 7.34, and 7.07 MPa, respectively. It can be observed that specimens with intermediate fractal dimensions maintain relatively low slopes in the exponential fitting curves over a wider stress range, indicating that the dominant effect of the exponential term is significantly delayed. As a result, these specimens exhibit relatively higher long-term strengths. In contrast, for specimens with excessively low or high fractal dimensions, the exponential term increases rapidly at relatively low stress levels, leading to an earlier acceleration of the steady-state creep rate and, consequently, a reduction in long-term strength:

(11) $${\dot{\varepsilon }}_{\mathrm{cp}}=A+Be^{C\sigma }$$

where *σ* is the creep stress, MPa; ${\dot{\varepsilon }}_{\mathrm{cp}}$ is the axial deformation rate during the steady-state creep stage corresponding to *σ*, in 10^−4^ mm/h; and A, B, and C are fitting coefficients.

#### 3.2.4. Burgers Model Fitting and Rheological Parameter Evolution

To further quantify the creep response of cemented coal gangue backfill with different aggregate mass fractal dimensions, a Burgers rheological model was introduced to fit the representative creep curves before macroscopic creep instability. The Burgers model consists of a Maxwell element and a Kelvin element connected in series, and can describe instantaneous elastic deformation, primary decelerating creep, and quasi-steady-state creep. Therefore, it is suitable for characterizing the main creep stages observed in the present stepwise accelerated creep tests.

Considering that the final accelerated creep stage is dominated by unstable crack coalescence and nonlinear damage evolution, it was not used for parameter identification. To ensure comparability among different aggregate gradation groups, the creep curves at 4 MPa were selected for Burgers model fitting. This stress level was below the creep instability threshold for all specimens and showed relatively stable time-dependent deformation, which is suitable for identifying the viscoelastic parameters of the specimens.

For the selected stress level, the Burgers model was expressed in the following equivalent form:

(12) $$\varepsilon \left(t\right)=\varepsilon_{0}+A\left(1-e^{t/\tau }\right)+Bt$$

where ε(t) is the axial creep strain at time t, $\varepsilon_{0}$ is the apparent initial strain at the selected stress level, A is the delayed elastic strain amplitude, τ is the retardation time, and B is the quasi-steady-state creep rate. In this expression, $\varepsilon_{0}$ reflects the instantaneous deformation level under the selected stress, A and τ describe the decelerating creep stage, and B represents the quasi-steady-state creep deformation rate.

To facilitate comparison among different fractal dimensions, the apparent instantaneous modulus $E_{0}$ and Maxwell viscosity coefficient $\eta_{M}$ were calculated as follows:

(13) $$E_{0}=\frac{\sigma }{\varepsilon_{0}/100}$$

(14) $$\eta_{M}=\frac{\sigma }{B/100}$$

where σ is the selected stress level, equal to 4 MPa in this fitting analysis. The nonlinear least-squares fitting and graphical analysis were performed using OriginPro 2021 (OriginLab Corporation, Northampton, MA, USA). The fitting results are summarized in Table 3.

The fitting results show that the Burgers-equivalent model can reasonably describe the creep curves at 4 MPa, with $R^{2}$ values ranging from 0.886 to 0.985. The relatively lower $R^{2}$ for the specimen with D=2.41 is mainly related to the small creep deformation amplitude and local fluctuation of the measured strain values at this stress level. Nevertheless, the fitted curve still captures the overall deformation trend.

The fitted parameters further reveal the influence of aggregate mass fractal dimension on creep resistance. The specimen with D=2.41 exhibited the lowest apparent initial strain $\varepsilon_{0}$ and the highest apparent instantaneous modulus $E_{0}$, indicating that this gradation had stronger resistance to instantaneous deformation under the same stress level. More importantly, the quasi-steady-state creep rate B reached the minimum value at D=2.41, while the corresponding Maxwell viscosity coefficient $\eta_{M}$ reached the maximum value. This indicates that the intermediate aggregate mass fractal dimension provided the strongest viscous resistance to time-dependent deformation among the tested gradation schemes.

In contrast, when the fractal dimension was either too low or too high, the fitted B value increased and the $\eta_{M}$ value decreased. For the low-fractal-dimension specimen, the higher creep rate may be attributed to the coarse-particle-dominated structure, larger inter-particle voids, and weaker matrix continuity. For the high-fractal-dimension specimen, excessive fine particles weakened the coarse aggregate skeleton effect and increased the amount of aggregate–matrix interface requiring cementitious bonding, making the specimen more susceptible to time-dependent deformation.

Based on the fitted Burgers-equivalent parameters, a short-term extrapolative prediction was further conducted under the assumption that the quasi-steady-state creep stage remained unchanged. The predicted strains at 24 h under 4 MPa were 1.516%, 1.322%, 1.592%, and 1.756% for specimens with D=2.20, 2.41, 2.59, and 2.79, respectively. Among them, the specimen with D=2.41 showed the lowest predicted creep strain, whereas the specimens with D=2.59 and D=2.79 exhibited greater time-dependent deformation. This result is consistent with the experimentally observed lower quasi-steady-state creep rate and higher empirical long-term strength of the intermediate-fractal-dimension specimen.

Therefore, the Burgers model fitting provides quantitative support for the conclusion that an appropriate aggregate mass fractal dimension can improve the creep stability of cemented coal gangue backfill. It should be noted that the above prediction is based on the fitted response of the 4 MPa creep curves and assumes that the quasi-steady-state creep rate remains stable during extrapolation. Therefore, it should be regarded as a comparative prediction of creep tendency rather than a direct prediction of long-term field deformation. Longer constant-stress creep tests are still required to further validate the long-term applicability of the fitted rheological parameters.

### 3.3. Acoustic Emission Characteristics During Creep Failure

**Figure 10:**
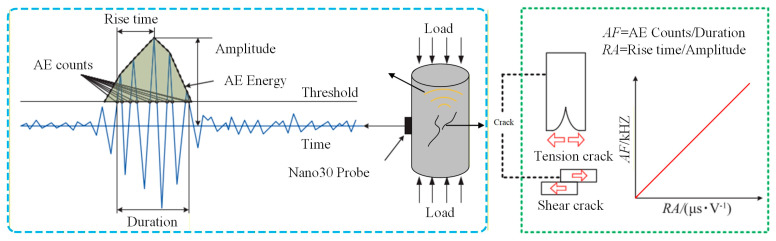
Schematic illustration of AE characteristic parameters and crack-mode classification. The arrows on the specimen indicate the axial loading direction; the arrows beside the crack schematics indicate tensile opening and shear sliding directions; and the connecting arrows indicate the analysis procedure from AE waveform parameters to RA–AF-based crack-mode classification.

During loading, the initiation, propagation, and coalescence of internal cracks are accompanied by the rapid release of strain energy, which propagates outward in the form of elastic waves. These elastic waves can be captured in real time by acoustic emission (AE) sensors, enabling dynamic monitoring of damage evolution and fracture processes in materials. Under creep loading conditions, AE activity is particularly sensitive to the evolution of the internal microstructure with time and stress level. As shown in Figure 10, typical AE parameters, including ring-down counts, AE energy, duration, rise time, and amplitude, were selected as the main indicators. Based on these parameters, characteristic indices such as RA and AF were constructed to distinguish different crack propagation modes. On this basis, the evolution of AE parameters during creep loading under different aggregate mass fractal dimensions was further analyzed to reveal the crack evolution mechanisms of cemented specimens during creep failure.

The RA–AF distribution was used to further reveal the cracking mechanism of cemented coal gangue backfill during creep failure. According to the normalized RA–AF classification criterion described in Section 2.3, AE events with relatively low normalized RA and high normalized AF values were classified as tensile cracking events, while those with relatively high normalized RA and low normalized AF values were classified as shear cracking events. It should be noted that the RA–AF method provides a semi-quantitative classification of AE events rather than direct visual observation of crack types. The reliability of this classification was improved by using normalized RA and AF values rather than fixed absolute thresholds, because the absolute values of RA and AF can be affected by sensor arrangement, threshold setting, coupling condition, specimen geometry, and wave attenuation. Nevertheless, the RA–AF classification still has limitations for cemented coal gangue backfill because AE signals may be influenced by aggregate–matrix friction, pore collapse, and particle rearrangement during creep loading. Therefore, the AE-based tensile/shear classification was not used as independent evidence alone, but was interpreted together with creep-rate evolution, macroscopic failure morphology, and SEM observations.

**Figure 11:**
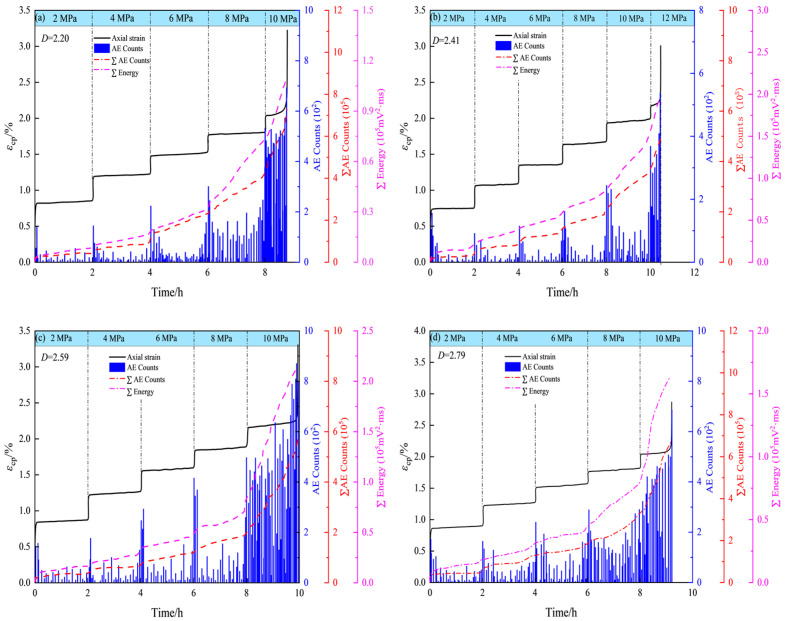
Relationships among AE ring-down counts (energy), axial strain, and time during the full creep process for specimens with different fractal dimensions: (**a**) D=2.20; (**b**) D=2.41; (**c**) D=2.59; (**d**) D=2.79.

Figure 11 shows the evolution of axial strain, ring-down counts, cumulative counts, and cumulative energy with time and loading level during the stepwise creep process for specimens with different aggregate mass fractal dimensions. For all fractal dimensions, the cumulative AE ring-down counts and cumulative AE energy exhibit an approximately exponential increase. Taking Figure 11b as an example, when the fractal dimension is *D* = 2.41, the ring-down count rate and energy rate increase sharply after the axial stress reaches 10 MPa, indicating that the specimen has entered a stage of unstable crack propagation, accompanied by accelerated damage evolution and highly active AE responses. Under other loading conditions, the AE activity exhibits distinct stage-dependent characteristics. During the instantaneous loading stage, friction between mineral particles, dislocation, and closure of pre-existing microcracks lead to relatively active AE responses. However, because no significant structural damage has yet occurred and damage evolution gradually stabilizes, both the creep rate and the AE ring-down count rate decrease continuously. During the steady-state creep stage, creep-induced damage is approximately balanced by compaction and crack-closure effects, resulting in relatively low and stable creep rates and ring-down count rates, and the AE activity enters a quiet period. With further loading, the specimen enters the accelerating creep stage, during which accumulated cracks propagate and coalesce on a large scale to form macroscopic fractures. Damage develops rapidly, and severe failure occurs within a short time, causing both the creep rate and the ring-down count rate to increase continuously until peak values are reached. For specimens with aggregate mass fractal dimensions of 2.20, 2.41, 2.59, and 2.79, the cumulative ring-down counts during creep loading were 7.27 × 10^5^, 4.98 × 10^5^, 5.87 × 10^5^ and 6.74 × 10^5^, respectively, while the corresponding peak ring-down counts were 627, 521, 875, and 712 Hz. At low stress levels, the creep rate is relatively low, and both AE ring-down counts and energy rate remain at low levels. As stress increases and creep damage accumulate, the creep rate accelerates and the AE signals become progressively stronger. At the 2–4 MPa stage, ring-down counts mainly appear as sparse pulses, and both cumulative AE counts and cumulative AE energy increase approximately linearly at a low rate. After entering the 6–8 MPa stage, the pulse frequency increases significantly, and the slopes of the cumulative curves increase progressively. At the 10 MPa stage, dense bursts of ring-down counts occur, and both cumulative AE counts and cumulative AE energy exhibit an obvious upward acceleration. This accelerated segment coincides with the rapid growth of axial strain at high stress levels, reflecting the transition of internal damage from stable propagation to rapid crack coalescence.

The fundamental reason for the differences in cumulative ring-down counts and energy accumulation under different fractal dimensions lies in the control exerted by aggregate gradation on the internal load-bearing skeleton and damage evolution paths of the cemented material. When the fractal dimension falls within a relatively optimal range, such as *D* = 2.41, the particle gradation is more favorable for forming a continuous and dense skeleton structure, leading to more uniform load transfer and relatively suppressed crack initiation and propagation. As a result, the AE ring-down counts are lower, the slopes of the cumulative curves are smaller, and energy release is more dispersed. In contrast, when the fractal dimension is either too low, with a high proportion of coarse particles and more pronounced pores and interfacial defects, or too high, with increased fine particles, weakened skeleton effects, and enlarged interfacial area, local stress concentration occurs more easily. In such cases, interfacial debonding and microcrack development accelerate rapidly at medium-to-high stress levels, resulting in a significant increase in AE event frequency and more concentrated energy release in the high-stress stage.

**Figure 12:**
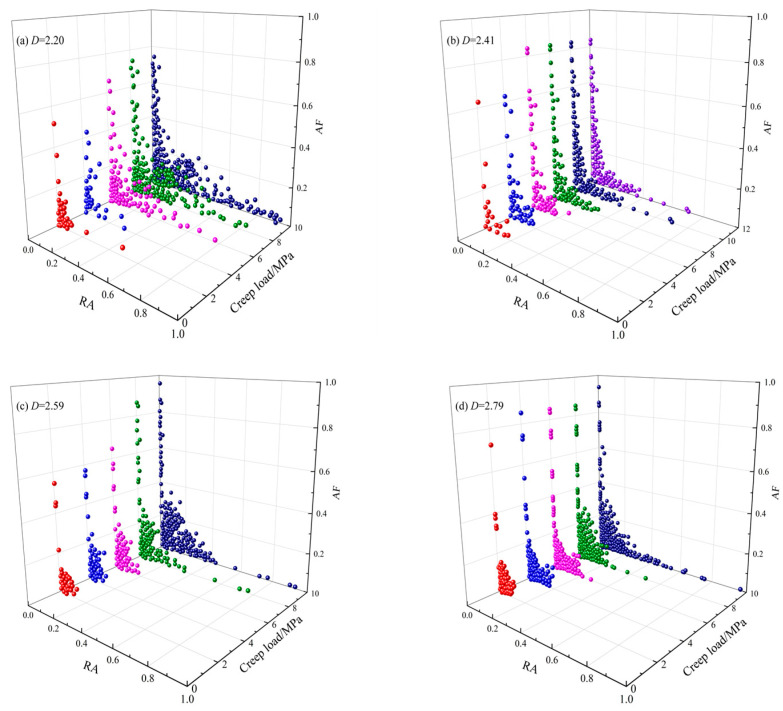
Normalized RA–AF scatter distributions during creep for specimens with different fractal dimensions: (**a**) D=2.20; (**b**) D=2.41; (**c**) D=2.59; (**d**) D=2.79. The colored dots represent AE events recorded at different creep stress levels: red, 2 MPa; blue, 4 MPa; magenta, 6 MPa; green, 8 MPa; dark blue, 10 MPa; and purple, 12 MPa. The 12 MPa stage applies only to the D=2.41 specimen.

Figure 12 presents the normalized RA–AF distributions throughout the creep process for specimens with different aggregate mass fractal dimensions. According to the normalized RA–AF criterion described in Section 2.3, AE events located in the low-$RA_{n}$/high-$AF_{n}$ region were classified as tensile cracking events, whereas those located in the high-$RA_{n}$/low-$AF_{n}$ region were classified as shear cracking events. At low stress levels, most data points are concentrated in the low-$RA_{n}$ region, indicating that crack activity is mainly associated with the initiation of tensile microcracks. As the stress level increases stepwise, the data points gradually extend toward the high-$RA_{n}$ region, and the number of shear cracking events increases significantly, especially at high stress levels where the distribution range becomes substantially broader. This feature indicates that, during creep damage evolution, the cracking mode gradually shifts from tensile-dominated AE activity to enhanced shear-related AE activity.

For the specimen with *D* = 2.41, the scatter distribution is relatively concentrated, with fewer events appearing in the high-RA region. Shear crack events mainly occur at high stress levels, suggesting that the internal structure remains relatively stable, interfacial sliding is restricted, and crack propagation is dominated by tensile cracking. In contrast, specimens with fractal dimensions of 2.20 and 2.79 exhibit a large number of high-RA events already at medium-to-high stress levels, with a significantly increased proportion of shear cracks and a more dispersed distribution range, indicating more active internal stress concentration and interfacial frictional sliding. The specimen with *D* = 2.59 shows an intermediate behavior, with a slightly higher number of shear crack events than the specimen with *D* = 2.41, but still lower than those with *D* = 2.20 and *D* = 2.79.

From the overall statistical results of the entire creep process, tensile cracks dominate in all specimens, whereas the proportion of shear cracks first decreases and then increases with increasing fractal dimension. The intermediate fractal dimension corresponds to the lowest proportion of shear cracks. This trend is consistent with the previously observed results for steady-state creep rate and long-term strength, indicating that when aggregate gradation falls within an optimal fractal range, the internal load transfer paths become more uniform, and interfacial sliding and shear propagation are suppressed. As a result, the transition from tensile-dominated damage to shear-dominated damage is delayed. Conversely, when the fractal dimension deviates from this range, structural heterogeneity increases, stress concentration intensifies, and shear cracks form and propagate more readily, ultimately leading to earlier creep instability.

**Figure 13:**
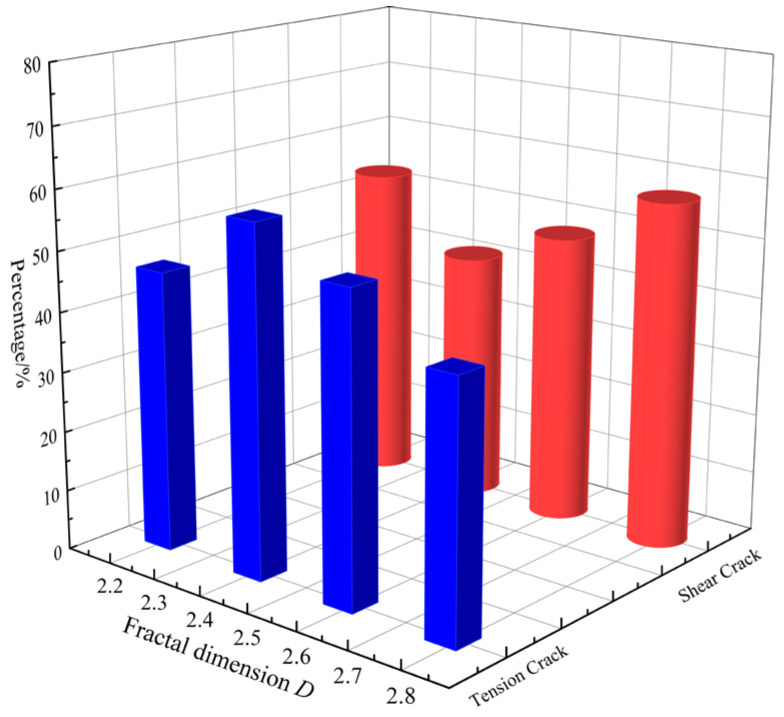
Proportions of AE events classified as tensile and shear cracking events under different fractal dimensions. Blue bars represent tensile cracking events, and red bars represent shear cracking events.

Based on the statistical results derived from Figure 12, the proportions of AE events classified as tensile and shear cracking events under different fractal dimensions were further calculated, as shown in Figure 13. A pronounced fractal-dimension effect can be observed in the relative proportions of the two cracking-event types. When D=2.20, the proportions of tensile and shear cracking events are 46.7% and 53.3%, respectively, indicating a slightly shear-dominated AE response. When the fractal dimension increases to 2.41, the proportion of tensile cracking events rises to 58.2%, while the proportion of shear cracking events decreases to 41.8%. This indicates that, under this fractal condition, the creep damage process is less dominated by unstable shear slip and crack coalescence, and the internal structure exhibits relatively better stability. When the fractal dimension further increases to 2.59, the proportions of tensile and shear cracking events become 51.5% and 48.5%, respectively, indicating a mixed tensile–shear cracking response. When the fractal dimension reaches 2.79, the proportion of shear cracking events increases to 57.7%, significantly exceeding that of tensile cracking events. This suggests that the increase in fine particle content enlarges the aggregate–matrix interfacial area and enhances interfacial sliding and friction, causing the AE response during creep failure to shift back toward shear dominance.

This phenomenon can be explained by the microstructural characteristics associated with different fractal dimensions. At a relatively low fractal dimension (*D* = 2.20), the proportion of coarse particles is relatively high, while the filling effect of fine particles is insufficient. As a result, the aggregate skeleton exhibits a discrete supporting structure, and the number of force chains formed between particles is limited and unevenly distributed. During creep loading, stress is mainly transferred through a few contact points, leading to strong local stress concentration. The interfacial transition zone (ITZ) therefore undergoes sliding and microdamage first. Since the interface is dominated by shear stress concentration, cracks tend to propagate along the aggregate–matrix interface, resulting in a higher proportion of shear cracks. When the fractal dimension increases to *D* = 2.41, coarse and fine particles form a more continuous load-bearing structure, load transfer paths become more uniform, and interfacial stress concentration is weakened. Under this condition, cracks are more likely to propagate in tension along the direction of the maximum principal stress, leading to a significantly reduced proportion of shear cracks. As the fractal dimension further increases, the fine particle content increases, the total interfacial area becomes larger, and the skeleton effect is weakened. Structural stability then depends more heavily on the cementitious matrix. Under sustained loading, interfacial sliding and shear dislocation become more frequent, resulting in an increased proportion of shear cracks once again.

### 3.4. Microstructural Damage Evolution Mechanism Controlled by Fractal Dimension

**Figure 14:**
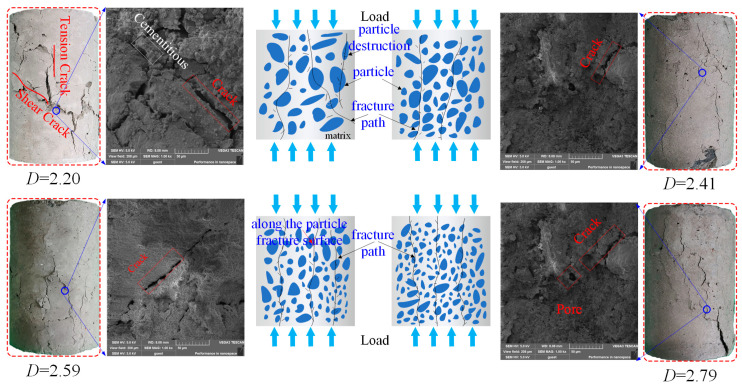
Macro–micro failure mechanisms under different fractal dimensions. Blue vertical arrows indicate the axial compressive loading direction; blue oblique connecting lines indicate the correspondence between selected macroscopic failure regions and SEM observation areas; black arrows identify particles, matrix regions, particle destruction, and fracture paths; and red boxes highlight representative cracks or pores.

To elucidate the governing mechanism by which aggregate mass fractal dimension controls the strength and creep instability behavior of cemented coal gangue backfill, a combined analysis was conducted based on macroscopic fracture morphology, scanning electron microscopy (SEM) observations, acoustic emission (AE) crack-mode statistics, and creep deformation characteristics (Figure 14). The results indicate that aggregate mass fractal dimension affects both the continuity of the particle skeleton and the integrity of the aggregate–matrix interface, thereby controlling crack initiation, crack propagation paths, and final failure modes. This mechanism can be interpreted from two coupled aspects: the load-bearing effect of the aggregate skeleton and the damage evolution of the interfacial transition zone (ITZ) between coal gangue particles and the cementitious matrix. Here, the skeleton effect refers to the load-bearing role of coarse aggregate particles and their contact network within the cementitious matrix, which helps transfer external stress and restrain matrix deformation under sustained loading.

From the perspective of macroscopic fracture morphology, specimens with relatively low and high fractal dimensions were more likely to form through-going cracks along local weak zones. For the specimen with *D* = 2.20, tensile and shear cracks coexisted, indicating that the coarse-particle-dominated structure provided partial skeleton support but also generated large inter-particle voids and weak interfacial regions. For the specimen with *D* = 2.79, the failure mode was more shear-dominated, suggesting that excessive fine particles weakened the coarse aggregate skeleton and promoted localized deformation. In contrast, the specimen with *D* = 2.41 exhibited more dispersed cracks with lower connectivity, and the failure mode was mainly characterized by tensile splitting. The specimen with *D* = 2.59 showed an intermediate tensile–shear composite failure mode. These observations are consistent with the AE statistical results. The shear crack proportions of the specimens with *D* = 2.20 and *D* = 2.79 were higher than those of the intermediate-fractal-dimension specimens, whereas the specimen with *D* = 2.41 showed the lowest shear crack proportion and the highest tensile crack proportion. This indicates that an appropriate aggregate gradation can suppress interfacial sliding and shear crack coalescence during creep loading. The normalized RA–AF classification therefore provides AE-based evidence for the proposed skeleton–interface coupling mechanism, in which the optimized aggregate gradation reduces shear-related damage evolution by improving skeleton continuity and aggregate–matrix bonding integrity.

Based on the combined SEM observations, AE crack-mode statistics, and creep deformation characteristics, the damage mechanisms under different aggregate mass fractal dimensions can be further interpreted as follows.

The proposed skeleton–interface mechanism is supported by the consistency among creep deformation, AE crack-mode statistics, macroscopic failure patterns, and SEM observations. For the specimen with D=2.41, the steady-state creep rate was relatively low, the empirical long-term strength was the highest, and the proportion of shear cracking events reached the minimum value of 41.8%. These macroscopic and AE characteristics are consistent with the SEM observations showing a more continuous aggregate skeleton, denser matrix filling, and fewer apparent interfacial defects. In contrast, specimens with D=2.20 and D=2.79 exhibited higher shear-cracking proportions, lower empirical long-term strengths, and earlier creep instability. These behaviors correspond to more pronounced inter-particle voids, weaker interfacial regions, and less continuous skeleton structures observed in the microstructural analysis. Therefore, the proposed mechanism is not inferred from SEM images alone, but from the coupled evidence of creep response, AE evolution, macroscopic fracture morphology, and microstructural observations. However, because the present study did not directly quantify three-dimensional pore connectivity, ITZ thickness, or local force-chain evolution, the proposed skeleton–interface mechanism should be interpreted as a mechanistic explanation supported by multi-scale experimental observations rather than as a fully quantified damage model.

For specimens with a relatively low fractal dimension, the high proportion of coarse particles resulted in larger inter-particle voids and a less continuous cementitious matrix. Although coarse particles could provide a certain skeleton support, the discontinuity of the matrix and the presence of large pores weakened the bonding between aggregates and hydration products. In this case, the ITZ was more prone to local debonding and sliding under sustained loading. As a result, cracks tended to initiate and propagate along the aggregate–matrix interface, promoting shear-type AE events and accelerating creep deformation.

For specimens with an intermediate fractal dimension, coarse particles formed a relatively continuous load-bearing skeleton, while fine particles filled the inter-particle voids and improved the compactness of the matrix. This gradation condition enhanced ITZ integrity and promoted more uniform stress transfer between the aggregate skeleton and the cementitious matrix. Consequently, crack propagation was more dispersed, and cracks were less likely to rapidly coalesce into through-going fractures. This explains why the specimen with *D* = 2.41 exhibited slower creep deformation, a lower proportion of shear cracks, higher compressive strength, and higher empirical long-term strength among the tested gradation schemes.

For specimens with a relatively high fractal dimension, excessive fine particles weakened the coarse aggregate skeleton effect and increased the specific surface area requiring cementitious bonding. Although fine particles can fill voids to some extent, excessive fine content may increase the number of weak interfaces and reduce the effectiveness of particle interlocking. This may lead to insufficient local bonding and a more heterogeneous ITZ, making the specimen susceptible to localized damage accumulation and interfacial shear cracking during high-stress creep. Therefore, the creep deformation accelerated more easily, and the specimen entered the creep instability stage earlier.

The above analysis also explains why the aggregate mass fractal dimension is more suitable for characterizing the gradation effect than conventional gradation descriptors. Conventional gradation descriptors, such as the maximum particle size, fine particle content, or coarse-to-fine aggregate ratio, can describe specific aspects of aggregate composition but cannot fully represent the continuous distribution characteristics of particles across the entire size range. For cemented coal gangue backfill, creep stability is controlled not only by the amount of coarse or fine particles, but also by the combined effect of skeleton continuity, void filling, and aggregate–matrix interfacial area. The aggregate mass fractal dimension integrates these gradation characteristics into a single quantitative parameter. Therefore, it can better explain the non-monotonic evolution of UCS, steady-state creep rate, empirical long-term strength, and tensile–shear cracking behavior observed in this study. This indicates that the fractal dimension is not merely a fitting parameter, but a structural index with clear physical meaning for evaluating the long-term stability of cemented granular backfill materials.

The favorable performance observed at D=2.41 is consistent with previous findings that a moderate aggregate gradation generally produces better mechanical performance than either excessively coarse or excessively fine gradations in cemented granular materials. However, unlike studies that used fine particle content, maximum aggregate size, or coarse-to-fine aggregate ratio as gradation descriptors, the present study expresses the gradation effect using aggregate mass fractal dimension. This allows the optimal gradation range to be linked not only to UCS, but also to creep rate, empirical long-term strength, AE crack-mode transition, and microstructural damage evolution.

From the perspective of damage evolution, when the fractal dimension deviates from the optimal range, interfacial defects and pores lead to earlier microcrack initiation. Meanwhile, interfacial frictional sliding and shear displacement intensify stress concentration at crack tips and accelerate crack propagation, enabling microcracks to coalesce more rapidly into macroscopic fractures. This process is reflected in the AE response as an increased proportion of shear cracks and a more concentrated release of AE events and energy at high stress levels. Therefore, the combined SEM and AE results support the interpretation that interfacial sliding and shear coalescence are the dominant damage mechanisms when the aggregate gradation is either too coarse or too fine.

In summary, the aggregate mass fractal dimension provides a unified explanation for both strength formation and creep failure mechanisms by regulating the continuity of the particle skeleton and the integrity of the interfacial structure. When the fractal dimension falls within an optimal range, the load-bearing skeleton is more continuous, interfacial defects are reduced, and stress distribution is more uniform. Under such conditions, cracks propagate mainly in a dispersed tensile manner, shear sliding is suppressed, and the material exhibits higher compressive strength, lower steady-state creep rate, and higher long-term strength. Conversely, when the fractal dimension deviates from this range, the interface becomes the dominant weak zone, shear sliding intensifies, and cracks coalesce rapidly, leading to reduced strength, deteriorated long-term stability, and earlier creep instability.

It should also be noted that the long-term performance of ecological cementitious materials may be affected not only by sustained mechanical loading, but also by aggressive environmental conditions such as acidic or alkaline mine water. Recent studies on sustainable cementitious composites incorporating agricultural waste have shown that accelerated aging in acidic and basic solutions can significantly affect durability-related properties [43]. Therefore, future studies should consider the coupled effects of aggregate gradation, sustained loading, and chemical erosion to evaluate the long-term durability of solid-waste-based backfill systems in aggressive underground environments. Although the present study focuses on the creep behavior of cemented coal gangue backfill under laboratory curing conditions, environmental durability may further influence the aggregate–matrix interface, pore structure, and long-term deformation resistance of ecological backfill materials. Therefore, future studies should consider the coupled effects of aggregate gradation, sustained loading, and chemical erosion to evaluate the long-term durability of low-carbon or solid-waste-based backfill systems in aggressive underground environments.

## 4. Conclusions

In this study, different coal gangue gradation schemes characterized by aggregate mass fractal dimension were designed based on fractal theory. Uniaxial compression tests, multi-stage loading creep tests, acoustic emission (AE) monitoring, and SEM analyses were conducted to systematically investigate the effects of aggregate mass fractal dimension on strength characteristics, long-term strength, and damage evolution mechanisms of cemented coal gangue materials. The main conclusions are as follows:

(1) Aggregate mass fractal dimension significantly affects both the strength characteristics and creep response of cemented coal gangue materials. Both P-wave velocity and uniaxial compressive strength show generally non-monotonic variations with increasing fractal dimension, and relatively high values are obtained within the intermediate fractal-dimension range. The UCS reaches its maximum value at D=2.41, indicating that this gradation provides the most favorable mechanical performance among the tested groups. The corresponding empirical long-term strengths for specimens with fractal dimensions of 2.20, 2.41, 2.59, and 2.79 are 6.62, 8.83, 7.34, and 7.07 MPa, respectively, further confirming that the intermediate fractal dimension improves long-term creep stability.

(2) The steady-state creep rate and crack evolution behavior are strongly regulated by the fractal dimension. An intermediate fractal dimension can significantly prolong the steady-state creep stage and suppress the onset of accelerating creep. AE-based RA–AF analysis shows that the proportion of AE events classified as shear cracking events decreases from 53.3% to 41.8% and then increases to 57.7% with increasing fractal dimension, demonstrating a transition from shear-dominated AE activity to tensile-dominated AE activity and then back to enhanced shear-related damage.

(3) Microstructural observations reveal that the fractal dimension governs creep instability by regulating skeleton continuity and interfacial integrity. At an intermediate fractal dimension, a more continuous load-bearing skeleton and a more intact interfacial structure are formed, resulting in more uniform stress distribution and predominantly tensile crack propagation. In contrast, when the fractal dimension is either too low or too high, interfacial defects and sliding become more pronounced, the proportion of shear cracks increases, and the material exhibits reduced strength and earlier creep instability.

(4) The main limitation of this study is that the creep behavior was evaluated using a stepwise accelerated creep loading scheme with a limited number of creep specimens. Therefore, the empirical long-term strength and Burgers-model-based prediction should be interpreted as comparative indices within the present experimental range rather than absolute long-term field values.

(5) Future studies should conduct more repeated creep tests and longer constant-stress creep tests to verify the statistical reliability and long-term applicability of the proposed fractal-dimension-based gradation optimization method. In addition, multi-scale techniques such as CT scanning, numerical modelling, and field-scale backfill monitoring can be used to further validate the skeleton–interface damage mechanism proposed in this study.

These conclusions indicate that aggregate mass fractal dimension can serve as an effective structural parameter linking aggregate gradation, creep deformation, and damage evolution of cemented coal gangue backfill. Compared with conventional gradation descriptors, the fractal dimension provides a more integrated representation of particle size distribution and can be used as a quantitative reference for optimizing aggregate gradation. The present results provide a theoretical and practical basis for improving the long-term stability of cemented coal gangue backfill and promoting the resource utilization of mining solid waste.

## Figures and Tables

**Table 1:** Mass fractions of the main chemical components of the raw materials, adapted from Ref. [37].

Materials	Compound Mass Fraction/%							
	SiO_2_	Al_2_O_3_	CaO	Fe_2_O_3_	MgO	Na_2_O	K_2_O	Others and LOI/%
Cement	21.60	9.58	54.31	3.25	2.10	0.30	0.65	8.21
Fly ash	52.19	26.49	6.12	7.18	1.87	0.89	2.11	3.15
Gangue	58.24	26.45	1.81	4.27	1.42	0.66	3.15	4.00

Note: Others and LOI were calculated by difference and include minor oxides and unquantified loss-on-ignition components.

**Table 2:** Complete mix design of cemented coal gangue backfill specimens.

Cement %	Fly Ash /%	Coal Gangue /%	Water /%	W/B	C/FA	Aggregate Condition	Solid Mass Concentration/%
16	4	65	15	0.75	4.0	Air-dried	85

Note: The four groups had the same binder–water proportion, aggregate condition, and solid mass concentration. Only the particle size distribution of coal gangue aggregate varied according to the designed aggregate mass fractal dimensions of *D* = 2.20, 2.41, 2.59, and 2.79.

**Table 3:** Burgers-equivalent fitting parameters of the 4 MPa creep curves.

*D*	*ε*_0_/%	*E*_0_/MPa	*A*/%	*B*/%·h^−1^	*τ*/h	*η*_M_/MPa·h	*R* ^2^
2.20	1.1825	338.3	0.0072	0.0136	0.0946	29,427.6	0.977
2.41	1.0554	379.0	0.0038	0.0110	0.0600	36,470.0	0.886
2.59	1.0999	363.7	0.0053	0.0203	0.0223	19,731.2	0.985
2.79	1.2198	327.9	≈0	0.0223	—	17,919.8	0.983

## Data Availability

The original contributions presented in this study are included in the article. Further inquiries can be directed to the corresponding author.
